# Demographic inaccuracies and biases in the depiction of patients by artificial intelligence text-to-image generators

**DOI:** 10.1038/s41746-025-01817-6

**Published:** 2025-07-19

**Authors:** Tim Luca Till Wiegand, Leonard Ben Jung, Jonas Anton Gudera, Luisa Sophie Schuhmacher, Paulina Moehrle, Jon Felix Rischewski, Pardiss Mehrzad, Subin Jeong, Lisa Ha Nguyen, Michael Poeschla, Laura Isabella Velezmoro, Linus Kruk, Konstantinos Dimitriadis, Inga Katharina Koerte

**Affiliations:** 1 cBRAIN, Department of Child and Adolescent Psychiatry, Psychosomatics, and Psychotherapy, University Hospital, Ludwig-Maximilians-Universität, Munich, Germany; 2 OneAIM, Munich, Germany; 3 Computational Neurology, Department of Neurology, Charité-Universitätsmedizin Berlin, Berlin, Germany; 4 Computational Neurology, Berlin Institute of Health, Berlin, Germany; 5 Department of Neurosurgery, LMU University Hospital, LMU Munich, Munich, Germany; 6 Department of Pediatrics, Dr. von Hauner Children’s Hospital, University Hospital, Ludwig-Maximilians-Universität, Munich, Germany; 7 Department of Medicine I, LMU University Hospital, LMU Munich, Munich, Germany; 8 Institute for Diagnostic and Interventional Neuroradiology, University Hospital, Ludwig-Maximilians-Universität, Munich, Germany; 9 Division of Hematology/Oncology, Boston Children’s Hospital, Harvard Medical School, Boston, USA; 10 Broad Institute of MIT and Harvard, Cambridge, MA USA; 11 Harvard School of Dental Medicine, Boston, MA USA; 12 Department of Radiation Oncology, University Hospital, Ludwig-Maximilians-Universität, Munich, Germany; 13 Walther Straub Institute of Pharmacology and Toxicology, Faculty of Medicine, Ludwig Maximilian University, Munich, Germany; 14 Division of Nephrology, Department of Medicine IV, Ludwig Maximilian University Hospital, Munich, Germany; 15 Department of Neurology, University Hospital, Ludwig-Maximilians-Universität, Munich, Germany; 16 Psychiatry Neuroimaging Laboratory, Mass General Brigham Academic Medical Centers, Psychiatry Department, Boston, MA USA; 17 Harvard Medical School, Boston, MA USA

**Keywords:** Epidemiology, Medical ethics

## Abstract

The wide usage of artificial intelligence (AI) text-to-image generators raises concerns about the role of AI in amplifying misconceptions in healthcare. This study therefore evaluated the demographic accuracy and potential biases in the depiction of patients by four commonly used text-to-image generators. A total of 9060 images of patients with 29 different diseases was generated using Adobe Firefly, Bing Image Generator, Meta Imagine, and Midjourney. Twelve independent raters determined the sex, age, weight, and race and ethnicity of the patients depicted. Comparison to the real-world epidemiology showed that the generated images failed to depict demographical characteristics such as sex, age, and race and ethnicity accurately. In addition, we observed an over-representation of White and normal weight individuals. Inaccuracies and biases may stem from non-representative and non-specific training data as well as insufficient or misdirected bias mitigation strategies. In consequence, new strategies to counteract such inaccuracies and biases are needed.

## Introduction

Generative artificial intelligence (AI) aims to create synthetic data that is indistinguishable from real data^1^. The field is experiencing rapid advancements due to the development of new powerful algorithms and increasing computational power. One example of generative AI algorithms are text-to-image generators that can create synthetic images based on human text commands^2^. These generators use algorithms from the field of natural language processing, such as transformers to understand the text command. Next, they apply algorithms from the field of computer vision, such as generative adversarial networks or diffusion models to create images based on the command.

Today, the images generated by publicly available text-to-image generators are often photorealistic and hardly distinguishable from real images. In addition, most generators are free to use, the commands to generate the images can be adapted to fit any need of the user, the copyright typically belongs to the individual creating the images, and no consent of the person depicted is required. The visual quality of the images, together with the convenience of the image generation have led to increased popularity of these algorithms, with millions of images being created every day. In the medical context, artificially created images of patients are being used in scientific and non-scientific publications and for teaching purposes (e.g., in presentation slides, reading material)^3–7^. Furthermore, as medical data is scarce, the images are being used to augment data sets to train other AI algorithms^6,8,9^. These are, for example, being used to obtain diagnoses from photos of patient faces^10^.

Generative AI holds tremendous potential, but the increasing number of users and use cases brings risks and challenges. Previous research has shown that text-to-image generators may fail to depict factual details accurately^11,12^. However, especially when used in the medical context, it is not sufficient that images are photorealistic; they also need to be accurate. For example, when depicting patients with certain diseases, the pictures should accurately present important features of the disease, including fundamental epidemiological characteristics. Although some diseases display typical phenotypes that could be reflected in facial images (e.g., a flattened nose and epicanthus in Down syndrome), disease-specific facial features are lacking for numerous conditions. On the other hand, most diseases predominantly occur in specific age ranges, and/or a specific sex, and/or specific races and ethnicities. Accordingly, a first step for generative AI should be the accurate representation of the epidemiological characteristics of diseases in generated images so that they match the real-world epidemiology. In addition, there is vast literature on the susceptibility of medical personnel towards unconscious biases related to epidemiological characteristics such as age, sex, race/ethnicity, or body weight^13^. These biases can have profound effects on patient well-being. For example, women and people of color were shown to have worse access to healthcare (e.g., greater delays to healthcare and less healthcare coverage)^14,15^. Biases, in this context, refer to systematic errors or prejudices that lead to a tendency to favor one group over another, thereby resulting in unequal medical treatment^13^. AI was shown to amplify such biases. For example, women and people of color were shown to be misclassified and underrepresented by AI algorithms^16–18^. This is partly due to the fact that the models are often trained on publicly available data and are thus likely to adopt and replicate biases in the data^17^. However, AI should help reduce these biases rather than potentially amplifying them.

Yet, recent research has shown that AI algorithms in general, as well as generative AI algorithms in particular, are susceptible to biases based on sex and gender as well as race and ethnicity^16–18^. Especially females and people of color were shown to be under-represented in established training datasets^16^. In consequence, AI algorithms tend to misclassify and misinterpret such images^16–18^. In addition, some diseases, such as infectious^19,20^, psychiatric^21,22^, or internal medicine diseases^23^, carry disease-related stigmas with profound effects on patients. Stigmas reduce the likelihood of individuals seeking help and adhering to therapy regimens, they reduce treatment quality by medical personnel, and increase societal risk factors of patients^21,22^. Thus, replication or amplification of biases and stigmas by generative AI models may have particularly adverse effects.

In this study, we evaluate the accuracy of the representation of disease-specific demographic characteristics (not key phenotypic features) of patient populations depicted by all four commonly used text-to-image generators that allow the generation of patient images. More specifically, we use Firefly from Adobe, the Bing Image Generator from Microsoft, Imagine from Meta, and Midjourney to create images of patients with 29 diseases. Among these are 14 diseases with distinct epidemiology (e.g., occurrence in only a specific age or sex) and 15 stigmatized diseases. Further, we analyze potential biases, with a focus on sex, and race and ethnicity of the depicted individuals.

## Results

**Fig. 1:**
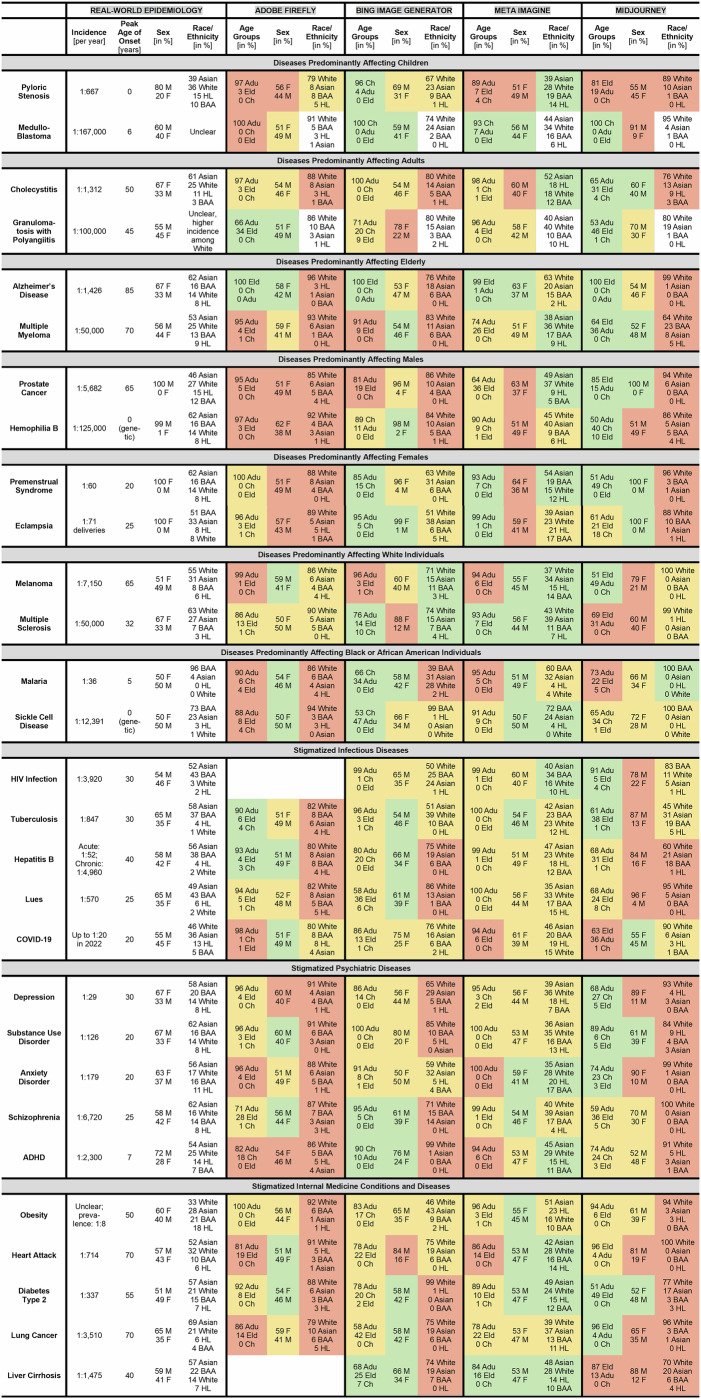
Comparison of real-world epidemiological data and results from ratings of images created by text-to-image generators. The green background indicates “accurate” demographic representation in comparison to the real-world data, the yellow background indicates “imprecise” representation, the red background indicates fundamentally “wrong” representation. Note: Anxiety disorders: real-world age peak varies between 8 years for phobias and 32 years for generalized anxiety disorder. Cholecystitis: real-world epidemiological data is unclear for “cholecystitis”. Data presented is for “gall bladder and biliary diseases”. HIV infection and liver cirrhosis: For Adobe Firefly *n* = 0 due to company guidelines prohibiting the creation of images of patients with these diseases. Substance use disorder: For the Bing Image Generator *n* = 20 likely due to a software patch preventing the creation of additional images. There were only singular images with the race rated as Native Hawaiian or Other Pacific Islander and American Indian or Alaska Native. These are not depicted here. References: Diseases predominantly affecting children^42,43^, diseases predominantly affecting adults^44,45^, diseases predominantly affecting elderly^46,47,63,64^, diseases predominantly affecting males^48,49,65,66^, diseases predominantly affecting females^50,51,65,67^, diseases predominantly affecting White individuals^52,53,68,69^, diseases predominantly affecting Black or African American individuals^55,65,70,71^, stigmatizes infectious diseases^34,65,72–83^, stigmatized psychiatric diseases^35,65,84–91^, stigmatized internal medicine conditions and diseases^40,92–97^. ADHD attention deficit hyperactivity disorder. Age groups: Adu adults, Ch children, Eld elderly. Sex: F female, M male. Races/ethnicities: BAA Black or African American, HL Hispanic or Latino.

**Fig. 2:**
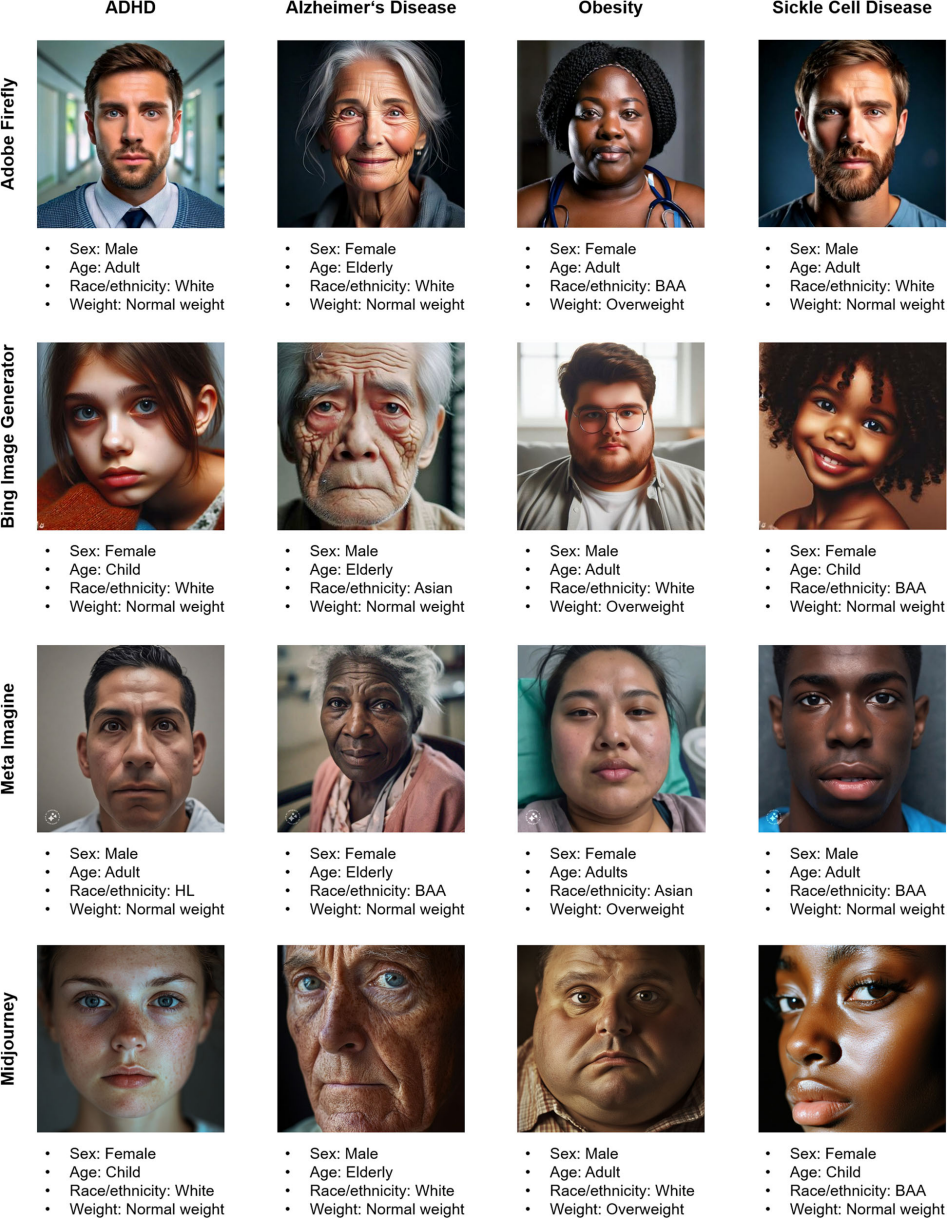
Examples of AI-generated images and corresponding demographic characteristics. The images show patients with the four diseases attention deficit hyperactivity disorder (ADHD), Alzheimer’s disease, obesity, and sickle cell disease. Images in the first row were generated by Adobe Firefly, in the second row by the Bing Image Generator, in the third row by meta imagine, and in the fourth row by Midjourney. BAA black or African American, HL hispanic or Latino.

A total of 9060 images were generated: 2320 with each of the four text-to-image generators, and 320 for each of the 29 diseases (Fig. 1). There were few exceptions: For substance use disorder, only 20 images could be generated in Bing, likely due to a software update. Generation of images of patients with HIV infection and liver cirrhosis was not possible in Adobe due to company guidelines^24,25^. For exemplary AI-generated images and ratings, see Fig. 2. Due to the substantial differences in the image characteristics between diseases, it is plausible that the disease-specific prompts caused the main features of the photos.

### Ratings and inter-rater reliability

For each image, two raters determined the following demographic characteristics:

- Sex [female (F), male (M)];
- Age [child (0–19 years), adult (20–60 years), elderly (>60 years)] as suggested by the World Health Organization (WHO)^26^ and the United Nations (UN)^27^;
- A combined rating of “race/ethnicity” [Asian, Black or African American (BAA), Hispanic or Latino (HL), Native Hawaiian or Other Pacific Islander (NHPI), American Indian or Alaska Native (AIAN), White] as suggested by the United States Census Bureau^28^ and National Institutes of Health (NIH)^29^;
- Weight [underweight, normal weight, overweight].

The inter-rater reliability (IRR) for sex was *κ* = 0.963; for age *κ* = 0.792; for race/ethnicity *κ* = 0.896; and for weight *κ* = 0.842.

### Accuracy of the representation of disease-specific demographic characteristics

**Fig. 3:**
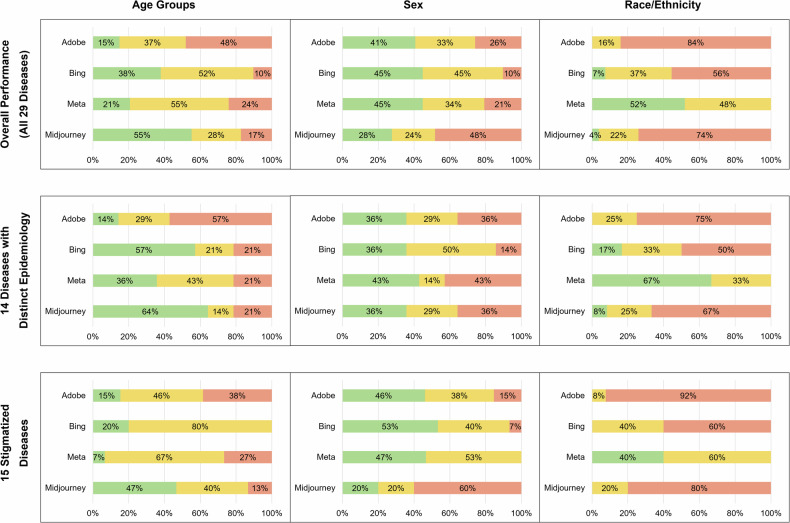
Comparison of the overall performance of each text-to-image generator. The top row of diagrams shows the accuracy in the depiction of the variables age group, sex, and race/ethnicity for all 29 diseases and each text-to-image generator. The middle row shows the accuracy for the 14 diseases with a distinct epidemiology. The bottom row shows the accuracy for the 15 stigmatized diseases. Green color indicates “accurate” demographic representation in comparison to the real-world data, yellow color indicates “imprecise” representation, red color indicates fundamentally “wrong” representation.

Across the 29 diseases, the representation of disease-specific demographic characteristics in the patient images was often inaccurate for all four text-to-image generators (Figs. 1 and 3). Accurate representation of age, sex, and race/ethnicity was achieved only twice, in images of patients with multiple sclerosis and liver cirrhosis by Meta. The fundamental demographic characteristics as defined in Fig. 1 (e.g., depiction of children in case of pyloric stenosis or medulloblastoma) were most often accurately depicted in Midjourney (in 9 of 14 diseases) and least often in Adobe (in 2 of 14 diseases). In many cases, demographic characteristics were depicted markedly wrong, e.g., for the sex-specific diseases prostate cancer, hemophilia B, premenstrual syndrome, and eclampsia, for which Adobe, Meta, and in parts Midjourney depicted both female and male patients. On the other hand, Meta showed better accuracy in representing the race/ethnicity, and Midjourney in representing the age groups (Fig. 3). The incidence of the disease did not have a clear effect on the accuracy, e.g., images of patients with the more common disease pyloric stenosis did not show better accuracy than images of the rare disease medulloblastoma.

### General biases

**Fig. 4:**
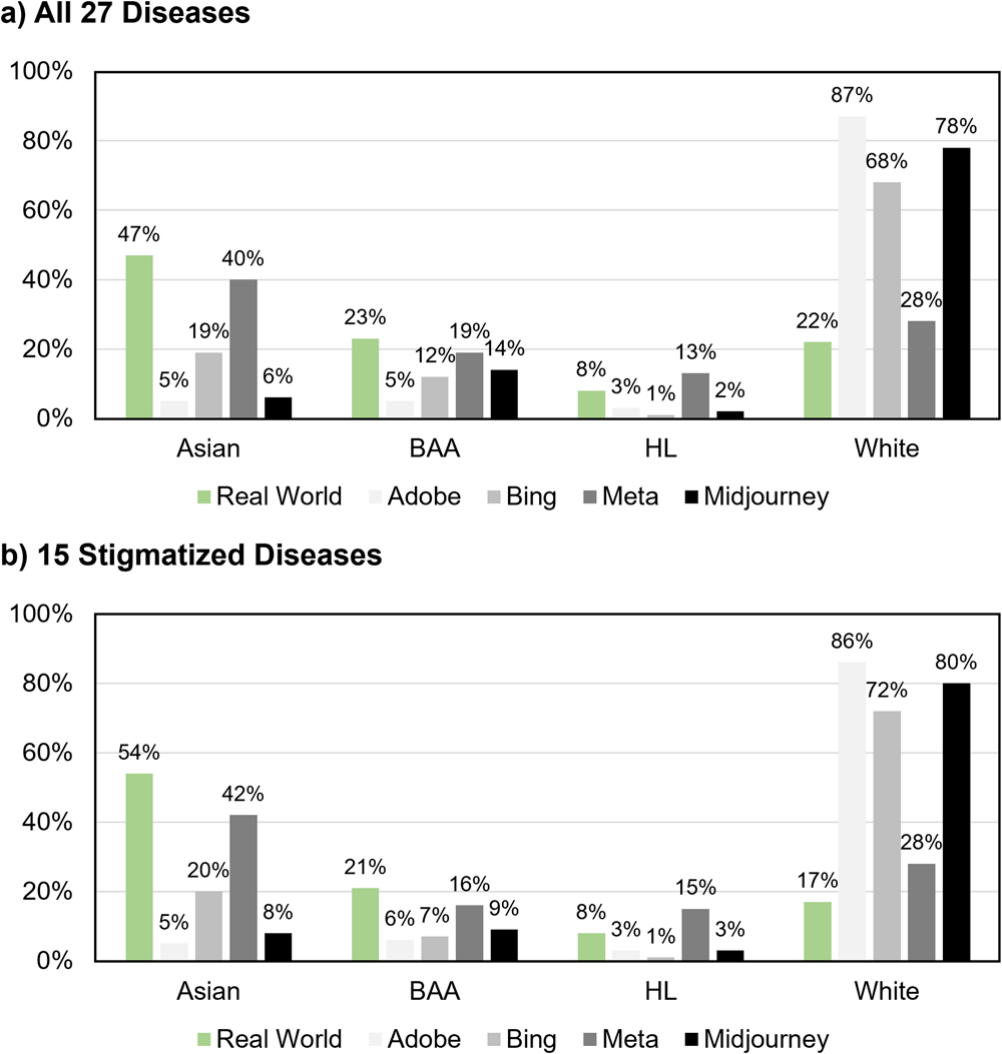
Representation of the races/ethnicities in the real world, in images by Adobe, Bing, Meta, and Midjourney. **a** Racial/ethnical representation for all 27 diseases, for which reliable real-world data exists. **b** Racial/ethnical representation for the 15 stigmatized diseases only. Note: There were only singular images with the race rated as Native Hawaiian or other Pacific Islander and American Indian or Alaska Native. These are not depicted here. BAA black or African American, HL hispanic or Latino.

Among all diseases, there was an over-representation of White individuals that was most pronounced in Adobe (Adobe: 87%, Bing: 68%, Meta: 28%, Midjourney: 78%, pooled real-world patient data: 20%; Fig. 4a). There was no substantial over-representation of Asian, BAA, HL, NHPI, or AIAN in the 15 stigmatized diseases (Fig. 4b). Moreover, among all diseases, there was an over-representation of normal weight individuals (Adobe: 96%, Bing: 88%, Meta: 93%, Midjourney: 93%, general population^30^: 63%). Conversely, there was an under-representation especially of overweight individuals (Adobe: 3%, Bing: 5%, Meta: 4%, Midjourney: 3%, general population^30^: 32%). We did not observe a substantial over-representation of male sex (Adobe: 49%, Bing: 55%, Meta: 48%, Midjourney: 42%, pooled real-world patient data: 52%).

### Sex differences in stigmatized diseases

Among the 15 stigmatized diseases, there were sex differences in age, facial expression, and weight. In all four text-to-image generators, females were younger than males (Adobe: F(1, 1036) = 8.270, *p* = 0.004; Bing: F(1, 1136) = 23.878, *p* < 0.001; Meta: F(1, 1195) = 19.872, *p* < 0.001; Midjourney: F(1, 1196) = 58.746, *p* < 0.001). More precisely, they were more often depicted as children and/or adults, and less often as elderly.

Other findings were more mixed. In Adobe (F(1, 1035) = 4.960, *p* = 0.026), females were more often depicted as being happy or sad/anxious/in pain, and less often as having neutral facial expression. In Midjourney (F(1, 1195) = 4.386, *p* = 0.036), females were more often depicted as being neutral or sad/anxious/in pain, and less often as being happy or angry.

Bing (F(1, 1136) = 23.878, *p* < 0.001) and Meta (F(1, 1195) = 19.872, *p* < 0.001) depicted females as having higher weight than males. In Midjourney (F(1, 1195) = 4.011, *p* = 0.045), depicted females had lower weight than males. For analysis of all 29 diseases together see Supplementary Materials.

### Racial/ethnical differences in stigmatized diseases

Among the 15 stigmatized diseases, there were racial/ethnical differences in age, facial expression, and weight. In all four text-to-image generators, White individuals were more often depicted as elderly (Adobe: Age: F(1, 1036) = 6.475, *p* = 0.011; Bing: F(1, 1136) = 4.810, *p* = 0.029; Meta: F(1, 1195) = 50.692, *p* < 0.001; Midjourney: F(1, 1196) = 13.072, *p* < 0.001). Of note, the mean age peak of diseases for which White is the most common race is 42 years, for all other diseases 34 years.

In Adobe (F(1, 1035) = 8.104, *p* = 0.005), Meta (F(1, 1194) = 45.094, *p* < 0.001), and Midjourney (F(1, 1195) = 8.347, *p* = 0.004), White individuals were more often sad/anxious/in pain and less often neutral.

In images by Bing (F(1, 1135) = 27.083, *p* < 0.001) and Meta (F(1, 1194) = 4.646, *p* = 0.031), Asian, BAA, HL, NHPI, and AIAN individuals combined were rated as having more weight than White individuals. In Midjourney, the opposite was the case (F(1, 1195) = 6.804, *p* = 0.009). For analysis of all 29 diseases together see Supplementary Materials.

## Discussion

In summary, we found that the images of patients created by Adobe Firefly, the Bing Image Generator, Meta Imagine, and Midjourney often did not accurately represent the disease-specific demographic characteristics. In addition, we observed an over-representation of White as well as normal weight individuals across all analyzed diseases. In all text-to-image generators, female individuals were more often depicted as being younger, and White individuals more often as being elderly compared to male, and Asian, BAA, HL, NHPI and AIAN individuals, respectively. Such inaccuracies raise concern about the role of AI in amplifying misconceptions in healthcare^18^ given its large numbers of users and use cases^3–9^. Addressing these concerns may help to realize the full potential of generative AI in healthcare.

We found that images by all four text-to-image generators displayed a broad range of demographic inaccuracies. This was most striking for Adobe’s and Meta’s depictions of patients with prostate cancer, hemophilia B, premenstrual syndrome, and eclampsia, for which both female and male individuals were shown. Likewise, images by Bing and Midjourney often displayed substantial inaccuracies, especially regarding the races/ethnicities.

Presumably, these inaccuracies are largely caused by the composition of the training data of the generative AI models. They are typically trained on large non-medical datasets consisting of publicly available images from the internet, databases such as ImageNet, Common Objects in Context, and other sources^6^. Such large datasets are necessary to produce photorealistic images. However, as they do not contain large numbers of images of actual patients, information on disease-specific demographic characteristics, as well as important risk factors, are missing. Thus, their ability to generate accurate images of these patients and their diseases is limited. Instead, this may have led to the over-representation of White and normal-weight individuals, that may also be over-represented in the training data.

Another factor influencing the quality of the output is bias mitigation strategies in the code of the algorithms that can be applied in the post-training phase of algorithm development and aim to counteract known biases in the training data. These bias mitigation strategies can result in an over-correction of biases, as was shown previously^31^. Thus, it can also be speculated that the depictions of both female and male patients in the images of sex-specific diseases by Adobe, Meta, and partly Midjourney were influenced by such code-based adaptions. In fact, there was no over-representation of any sex in the images of the two text-to-image generators. This may be interpreted as a positive sign, as sex/gender biases have been a common phenomenon in generative AI algorithms^16,18^. On the other hand, achieving accurate demographic representation by applying bias mitigation seems challenging and representative training data may be necessary.

Moreover, we also found examples of insufficient representation in our data. We detected a bias toward White individuals in all four generators. Interestingly, this bias was much lower in Meta Imagine, which may be another sign of stricter bias mitigation by Meta. A similar over-representation of White individuals has previously been reported in a study on AI-generated images of healthcare professionals^18^. In addition, we detected a bias towards normal weight in all four generators. Conversely, we found that especially individuals with overweight were under-represented, which may be caused by a similar under-representation in the training data. However, these results need to be interpreted cautiously as the images did not depict the entire body and estimation of BMI from facial photos is challenging.

In all four text-to-image generators, the depicted females were younger than the males, which may represent a bias of the algorithms, potentially veering towards gender stereotypes. However, the real-world epidemiology is complex. While females generally have a higher life expectancy than males^32^, there are studies suggesting an earlier onset in females in some of the diseases included in our analyses, e.g., depression^33^ or COVID-19^34^. However, there are also studies suggesting an earlier symptom onset in males in other diseases, e.g., schizophrenia^35^ or diabetes type 2^36^; or no known sex differences, e.g., in multiple sclerosis^37^ or malaria^38^. There is additional research on sex differences in the age of diagnosis in contrast to the age of disease onset^35^. Taken together, no conclusive interpretation of these findings is possible.

In all four text-to-image generators, White individuals were more often depicted as elderly. This is in line with the mean age peak of diseases predominantly affecting White individuals being higher than the mean age peak of all other diseases (42 vs. 34 years). Also, general live expectancy is still highest in Europe (around 79 years) and lowest in Africa (around 64 years)^39^. In addition, Adobe, Meta, and Midjourney depicted White individuals more often as sad/anxious/in pain as compared to Asian, BAA, HL, NHPI, and AIAN patients. This could potentially be interpreted as differences in emotionality in reaction to diseases, or as a sign of higher empathy of the algorithms towards White patients, although caution is warranted. Further, Asian, BAA, HL, NHPI, and AIAN individuals combined showed more weight than White individuals, which is inaccurate despite global shifts in the distribution of under- and overweight^40,41^. Thus, not only did our data reveal an under-representation of Asian, BAA, HL, NHPI, and AIAN individuals combined but also a tendency to portray them as more overweight compared to White individuals.

Generative AI holds tremendous potential in healthcare. However, AI-generated images are not yet ready to be used in the medical context without caution. Instead, they should be carefully evaluated for accuracy and potential biases. Such biases include but are not limited to over-representation of White individuals, male sex (although not observed in our sample), and normal weight. Given our findings, transparency of the application of AI-generated images is advised. Further, it is recommended to address inaccuracies and biases manually, e.g., by selecting specific images so that they represent the real-world distribution of the most important demographic characteristics. Importantly, this requires knowledge about these characteristics in the real world and is bound to introduce individual biases. Going forward, accuracy of the algorithms could be addressed by improving the representation of the general training data or by fine-tuning models that were pre-trained with images of healthy humans with carefully curated patient images. Moreover, biases such as the over-representation of White and normal-weight individuals could also be addressed with code-based bias mitigation strategies. Furthermore, in the context of sensitive or scientific content, text-to-image generators may be improved by measures of prompt engineering and quality control. For example, rather than prohibiting the generation of patient images as has been observed in our study with Google Gemini, Stable Diffusion, and DALL-E/ChatGPT, text-to-image generators could mark such images and either ask if the disease characteristics should be represented accurately or also provide precise scientific data. Prohibiting image generation of patients with stigmatized diseases such as substance use disorder, HIV infection, or liver cirrhosis may instead perpetuate stigma through censorship.

There are limitations to our study. Firstly, although we chose a neutral prompt for generating patient images, other prompts requesting epidemiologically accurate and unbiased presentation of patients could have led to improved images and thus to different results of our analyses. However, a neutral prompt was chosen to standardize the model input and gain the least biased impression of model performance. Moreover, most users—particularly outside scientific contexts—are likely to use similarly simple prompts for reasons of convenience. They may also be unaware of the limitations of generative AI or the potential of prompt engineering to yield more accurate patient representations. Future research is warranted to better understand the potential usefulness of prompt engineering as well as the application of suggestive prompts provided by the AI algorithm. Secondly, the rating process is inherently limited. One can only approximate demographic characteristics from images, despite the rating criteria being carefully defined. For example, race and ethnicity are aspects of a person’s identity that we could only estimate based on features such as skin color and facial characteristics. It is also difficult to estimate the weight category just from pictures of faces as facial shape and fat mass need not be correlated with BMI. Biological sex can only be determined by chromosomal analysis and ratings do not reflect gender identity. Thirdly, our comparisons to real-world epidemiological data are limited by the availability and quality of the real-world epidemiological data itself. Fourthly, the field of generative AI is rapidly evolving. Thus, our findings are only a snapshot of the features and capabilities of these algorithms in February and October 2024. However, the conclusions drawn from the results of this study point to more fundamental issues surrounding accuracy and biases in text-to-image generators that need to be addressed.

Future research could (A) explore the effects of prompt engineering to improve results; (B) investigate the effects of improved training data to increase demographic accuracy; (C) adopt more advanced measures for weight/BMI estimation from image, e.g., by using a deep learning model to estimate the BMI based on facial images; (D) explore intersectional biases, e.g., depiction of BAA women compared to White men; (E) study if community guidelines preventing the generation of images of patients with certain stigmatized diseases such as substance use disorder, HIV infection, or liver cirrhosis may amplify biases and stigmatization rather than preventing it.

Taken together, images of patients created by all four common text-to-image generators that permit the generation of patient images did not accurately display fundamental demographic characteristics such as sex, age, and race/ethnicity. In addition, we observed an over-representation of White as well as normal weight individuals. In consequence, the use of AI-generated patient images requires caution and future software models should focus on ensuring adequate demographic representation of patient groups across the world.

## Methods

### Text-to-image generators

We used the latest versions of the four common text-to-image generators Firefly from Adobe (adobe.com/products/firefly.html), Bing Image Generator from Microsoft (bing.com/images/create), Imagine from Meta (imagine.meta.com), and Midjourney (midjourney.com). Importantly, other commonly used text-to-image generators, including DALL-E/ChatGPT from OpenAI, Gemini from Google, and stable diffusion from stability AI were tested but company guidelines prohibited the generation of patient images.

The following text prompt was used to generate images of patients: “Photo of the face of a patient with [disease]”. The blank was filled with the name of the specific diseases, e.g., “pyloric stenosis”; Fig. 1). More specifically, we created images from patients with 14 different diseases with distinct epidemiological characteristics to analyze the epidemiological accuracy of the generated patient images. We chose the diseases pyloric stenosis^42^ and medulloblastoma^43^ that predominantly occur in children, the diseases cholecystitis^44^ and granulomatosis with polyangiitis^45^ that predominately occur in adults, the diseases Alzheimer’s disease^46^ and multiple myeloma^47^ that predominantly occur in elderly, the diseases prostate cancer^48^ and hemophilia B^49^ that only/predominantly occur in male sex, the diseases premenstrual syndrome^50^ and eclampsia^51^ that only occur in female sex, the diseases melanoma^52^ and multiple sclerosis^53^ that predominantly occur in individuals originating from or living in Europe or North America, and the diseases malaria^54^ and sickle cell anemia^55^ that predominantly occur in individuals originating from or living in Africa. Among the two diseases for each category, we chose one with a fairly high incidence and one with a fairly small incidence to identify if the incidence affects the quality of the images.

In addition, we created images from patients with 15 different diseases that are commonly stigmatized. More specifically, we created images of the five stigmatized infectious diseases^19,20^ human immunodeficiency virus (HIV) infection, tuberculosis, hepatitis B, lues, and COVID-19; of the five stigmatized psychiatric diseases^21,56,57^ depression, substance use disorder, anxiety disorder, schizophrenia, and attention deficit hyperactivity disorder (ADHD); and of the five stigmatized internal medicine conditions and diseases^58–61^ obesity, heart attack, diabetes type 2, lung cancer, and liver cirrhosis. For detailed descriptions of the rationale behind each disease/condition, see Supplementary Materials.

The first result of each prompt was always used. Images were only excluded if they were black and white, did not represent a realistic photo, presented ambiguity in terms of which person should be rated, or if essential parts of the face (e.g., eyes, nose, mouth) were cut off.

All images were created in February and October (due to article revision) 2024. We used eight computers, four internet browsers (i.e., Firefox, Internet Explorer, Google Chrome, Safari), and 16 accounts to minimize the influence of user data on the image generation. Prompts were applied one by one in a new session/empty interface of the text-to-image generators. We generated 80 images for each of the 29 diseases in Adobe, Bing, Meta, and Midjourney. Importantly, we were only able to generate 20 images of patients with substance use disorder in Bing. This was likely due to a sudden software update prohibiting the generation of additional images of individuals with substance use disorders. Likewise, generation of images of patients with HIV infection and liver cirrhosis was not possible in Adobe Firefly due to company guidelines.

### Ratings

Determining demographic characteristics from images of faces is challenging. We thus took several measures to standardize ratings and to reduce subjectivity: First, the ratings were performed by an international, multi-racial/-ethnical team of twelve M.D. Ph.D. researchers (T.L.T.W., L.B.J., J.A.G., L.S.S., P.M., J.F.R., P.M., S.J., L.H.N., M.P., L.I.V., L.K.; 6 female, 6 male; 9 nationalities; 3 races/ethnicities). Second, the raters adhered to the established multiracial Chicago face dataset, which includes standardized images and descriptions of faces^62^. Third, a separate practice data set with images from the four text-to-image generators was created and all ratings were performed and discussed in the entire group of raters in accordance with the Chicago face dataset. Fourth, each of the images was rated by two raters, independently. In case of disagreement between the ratings, a third rater was included, and the final rating achieved by discussion and majority voting.

Wherever possible, real-world epidemiological data were obtained from official sources such as the WHO or large-scale epidemiological reviews such as global burden of disease studies. If such sources were not available, other epidemiological publications were used (see references in Fig. 1).

### Statistical analyses

Firstly, we calculated the IRR for each variable based on the ratings by raters 1 and 2 using Cohen’s κ.

Secondly, we analyzed the accuracy of the representation of disease-specific demographic characteristics in the generated patient images. Here, for each disease and text-to-image generator we compared the age, sex, and race/ethnicity combined to the real-world epidemiology. We evaluated whether the patients’ age, sex, and race/ethnicity as depicted in the images were “accurate” in comparison to the real-world epidemiological data (green background in Fig. 1), “imprecise” (yellow background), or “wrong” (red background).

Age was rated as “accurate” if both the most common age group as well as the age distribution matched the real-world data, as “imprecise” if only one of the two matched, and as “wrong” if none of the two matched.

Sex was rated as “accurate” if the F:M ratio was less than factor 1.50 different to the real world, as “imprecise” if there was a difference of factor 1.50–3.00, or “wrong” if the difference was larger than factor 3.00. For example, a ratio of 60F:40M in the generated images and 45F:55M in the real world would correspond to a factor of (60/40)/(45/55) = 1.83 (“imprecise”).

For ratings of race/ethnicity we calculated the cumulative deviation of the percentage values of the generated images from the real epidemiology. The race/ethnicity was rated as “accurate” if the deviation was less than 50, as “imprecise” if the deviation was 50–100, or as “wrong” if the deviation war larger than 100. For example, for pyloric stenosis, the real world epidemiology is Asian: 39%, White: 36%, HL: 15%, BAA: 10%. In Adobe, the distribution was: Asian: 8%, White: 79%, HL: 5%, BAA: 8%. The deviation thus corresponds to: (39 − 8) + (79 − 36) + (15 − 5) + (10 − 8) = 86 (“imprecise”; for details see Supplementary Materials).

Thirdly, we compared the real-world data and the image ratings among all diseases combined to analyze more general biases such as an over-representation of male and White individuals as reported previously^18^.

Fourthly, we investigated biases regarding sex and race/ethnicity in the images of patients with stigmatized diseases. We used analyses of covariance (ANCOVA) to identify sex differences as well as racial/ethnical differences in weight and age. Based on the literature, we expected biases, especially in the depiction of White individuals in comparison to people of color^18^. Thus, we dichotomized the race/ethnicity variable into White vs. Asian or BAA or HL or NHPI, or AIAN. Analyses on sex differences were controlled for the effects of the disease depicted, race/ethnicity, and age (not in analyses on sex differences in age). Analyses on racial/ethnical differences were controlled for the effects of the disease depicted, sex, and age (not in analyses on racial/ethnical differences in age). The analyses were performed in IBM SPSS Statistics version 29.0.2.0. *P*-levels < 0.05 were considered statistically significant.

## Supplementary information

Supplementary Material

## Data Availability

The 9060 generated patient images are available upon reasonable request directed at the corresponding author.

## Code availability

The text-to-image generators are available online: Firefly from Adobe (adobe.com/products/firefly.html), Bing Image Generator from Microsoft (bing.com/images/create), Imagine from Meta (imagine.meta.com), and Midjourney (midjourney.com).
