# Supplementary material for: Demographic inaccuracies and biases in the depiction of patients by artificial intelligence text-to-image generators
Source: NPJ Digit Med. 2025 Jul 19;8:459. doi: 10.1038/s41746-025-01817-6 (PMC12276360; doi:10.1038/s41746-025-01817-6)
Supplement: Supplementary file 1 — Supplementary Material [file 41746_2025_1817_MOESM1_ESM.pdf]

SUPPLEMENTARY MATERIALS

Rationale for the Choice of Diseases

A total of 29 diseases were analyzed. While many diseases show distinct epidemiological patterns or are associated with stigma, the rationales for the choices of the 29 diseases analyzed are outlined in the following.

Diseases predominantly affecting certain demographic groups:

- Diseases predominantly affecting children:
  - Pyloric stenosis[1]:
    - Peak age of onset: 0 years
    - Fairly high incidence: 1:667 /year
  - Medulloblastoma[2]:
    - Peak age of onset: 6 years
    - Fairly low incidence: 1:167,000 /year
- Diseases predominantly affecting adults:
  - Cholecystitis[3]:
    - Peak age of onset: 50 years
    - Fairly high incidence: 1:1,312 /year
  - Granulomatosis with polyangiitis[4]:
    - Peak age of onset: 45 years
    - Fairly low incidence: 1:100,000 /year
- Diseases predominantly affecting elderly:
  - Alzheimer's disease[5-7]:
    - Peak age of onset: 85 years
    - Fairly high incidence: 1:1,426 /year
  - Multiple myeloma[8]:
    - Peak age of onset: 70 years
    - Fairly low incidence: 1:50,000 /year
- Diseases predominantly affecting males:
  - Prostate cancer[9-11]:
    - Sex distribution: 100 M, 0 F
    - Fairly high incidence: 1:5,682 /year
  - Hemophilia B[12]:
    - Sex distribution: 99 M, 1 F

        - Fairly low incidence: 1:125,000 /year
- Disease predominantly affecting females:
    - Premenstrual syndrome[10,13,14]:
        - Sex distribution: 100 F, 0 M
        - Fairly high incidence: 1:60 /year
    - Eclampsia[15]:
        - Sex distribution: 100 F, 0 M
    - In addition: Image generation prohibited by Bing using “Vulvar cancer”, or “Breast cancer”, image generation prohibited by Meta using “Mammary cancer”, “Breast cancer”, or “Endometriosis”
- Diseases predominantly affecting white individuals:
    - Melanoma[16,17]:
        - Racial/ethnical distribution: 55 White, 31 Asian, 8 BAA, 6 HL
        - Fairly high incidence: 1:7,150 /year
    - Multiple sclerosis[18,19]:
        - Racial/ethnical distribution: 63 White, 27 Asian, 7 BAA, 3 HL
        - Fairly low incidence: 1:50,000
- Diseases predominantly affecting black or African American individuals:
    - Malaria[10,20]:
        - Racial/ethnical distribution: 96 BAA, 4 Asian, 0 HL, 0 White
        - Fairly high incidence: 1:36 /year
    - Sickle cell disease[10,21,22]:
        - Racial/ethnical distribution: 73 BAA, 23 Asian, 3 HL, 1 White
        - Fairly low incidence: 1:12,391
    - In addition: Image generation prohibited by Bing using “Ebola”

<u>Stigmatized diseases</u>:
- Stigmatized infectious diseases[23,24]:
    - HIV infection[10,25-28]:
        - Fairly high incidence: 1:3,920 /year
        - Reason for frequent stigmatization: Sexually transmitted disease; association with marginalized groups (e.g., men who have sex with men, sex workers, drug users), contagiousness and fatality

        - In addition: “HIV” often used; image generation prohibited by Bing using “AIDS” instead of “HIV”
    - Tuberculosis[10,29-31]:
        - Fairly high incidence: 1:847 /years
        - Reason for frequent stigmatization: Association with poverty, contagiousness and fatality
    - Hepatitis B[10,32-34]:
        - Fairly high incidence: Acute: 1:52 /year, chronic: 1:4,960 /year
        - Reason for frequent stigmatization: Sexually transmitted disease; association with marginalized groups (e.g., sex workers, drug users), contagiousness and fatality
    - Lues[35-37]:
        - Fairly high incidence: 1:570 /year
        - Reason for frequent stigmatization: Sexually transmitted disease; association with marginalized groups (e.g., men who have sex with men, sex workers), contagiousness
        - In addition: Image generation prohibited by Bing and Meta using “Syphilis” instead of “Lues”
    - COVID-19[38-42]:
        - Fairly high incidence: Up to 1:20 /year in 2022
        - Reason for frequent stigmatization: Associated with fear of contagion, misinformation, and xenophobia, particularly early in the pandemic
- Stigmatized psychiatric diseases[43-45]:
    - Depression[10,44,46-48]:
        - Fairly high incidence: 1:29 /year
        - Reason for frequent stigmatization: Belief that the disease signifies personal weakness, and lack of resilience
    - Substance use disorder[10,43,49-51]:
        - Fairly high incidence: 1:126 /year
        - Reason for frequent stigmatization: Belief that disease signifies moral failing, and lack of willpower; association with homelessness
        - In addition: Image generation prohibited by Bing using “Drug addiction” instead of “Substance use disorder”
    - Anxiety disorder[10,45,46,52]:

        - Fairly high incidence: 1:179 /year
        - Reason for frequent stigmatization: Belief that the disease signifies weakness, and exaggerated sensitivity
    - Schizophrenia[10,44,46,53]:
        - Fairly high incidence: 1:6,720 /year
        - Reason for frequent stigmatization: Public fears about unpredictability and potential violence of patients
    - ADHD[10,46,54,55]:
        - Fairly high incidence: 1:2,300 /year
        - Reason for frequent stigmatization: Belief that the disease leads to/is an excuse for bad behavior, reduced academic efforts, and abuse of medication
- Stigmatized internal medicine conditions and diseases[56-59]:
    - Obesity[60-63]:
        - Fairly high incidence/prevalence: Incidence unclear, prevalence: 1:8
        - Reason for frequent stigmatization: Belief that the disease signifies moral failing, and lack of willpower; contradiction to common beauty ideals
    - Heart attack[10,59,64]:
        - Fairly high incidence: 1:714 /year
        - Reason for frequent stigmatization: Belief that the disease signifies unhealthy lifestyle/diet, and lack of willpower
    - Diabetes type 2[10,56,65]:
        - Fairly high incidence: 1:337 /year
        - Reason for frequent stigmatization: Belief that the disease signifies unhealthy lifestyle/diet, and lack of willpower
    - Lung cancer[10,57,66]:
        - Fairly high incidence: 1:3,510 /year
        - Reason for frequent stigmatization: Association with smoking; belief that the disease signifies unhealthy lifestyle, and lack of willpower
    - Liver cirrhosis[10,58,67]:
        - Fairly high incidence: 1:1,475 /year
        - Reason for frequent stigmatization: Association with alcohol abuse; belief that the disease signifies unhealthy lifestyle, and lack of willpower

Comparing Race/Ethnicity in the Generated Images to the Real-World Epidemiology

Based on the image ratings, we determined *how common a race or ethnicity is in a disease* according to the text-to-image generators. For example, in images generated by Bing, 67% of patients with pyloric stenosis were White, 23% Asian, 9% BAA, and 1% HL (**Table 1**). In contrast, however, real-world epidemiological studies most commonly report the incidence, i.e., *how common a disease is in a race or ethnicity*. To allow for comparison between the generated images and the real-world data, we thus calculated *how common a race or ethnicity is in a disease* in the real world. To this end, we first collected the real-world incidences for the races/ethnicities for each disease. As incidences for Native Hawaiian or Other Pacific Islander or American Indian or Alaska Native are widely lacking, we focused on the four categories Asian, BAA, HL, and White. Based on these real-world incidences, we calculated the percentage distributions and interpolated these percentages based on the real-world number of Asian, BAA, HL, and White individuals (shown in **Table 1**).

Facial Expression in the Generated Images

In addition to the variables sex, age, race/ethnicity, and weight, we rated the facial expression of the persons depicted [neutral, happy, angry, sad or anxious or in pain, other]. Among all four generators, patients with anxiety disorders, depression, and heart attack were most often depicted as sad, anxious, or in pain. This seems appropriate given the cardinal symptoms of these diseases. On the other hand, patients with medulloblastoma, multiple myeloma, and prostate cancer were among those most often depicted as happy. The reasons for this are unclear as all three cancers are still serious conditions that may lead to death.

Sex Differences in Age, Facial Expression, and Weight Across all 29 Diseases

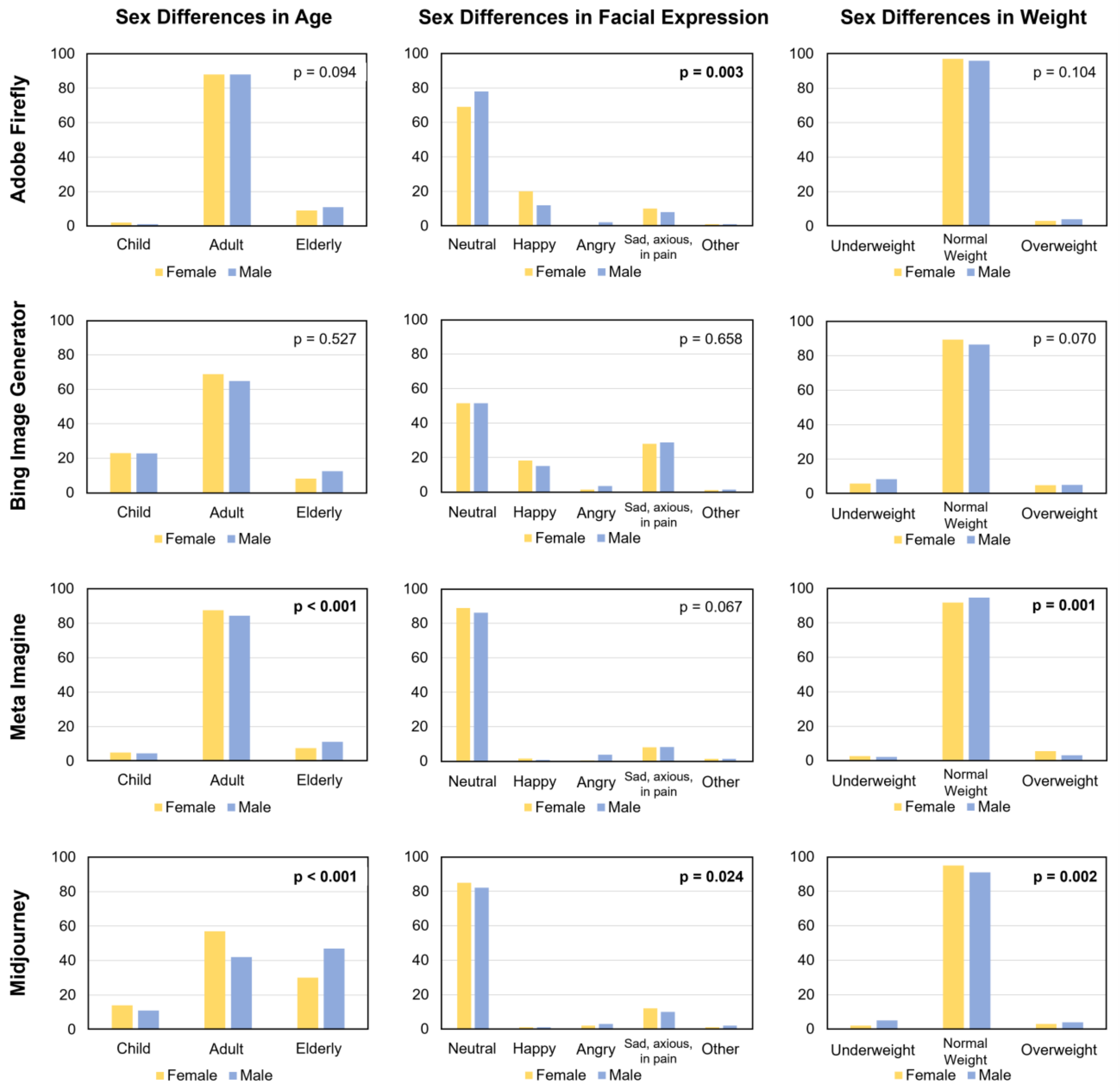

***Supplementary Figure 1*: Sex differences in age (left), facial expression (middle), and weight (right) across all diseases**. The first row of graphs shows the sex differences for images created by Adobe, the second row for images by Bing, the third row for images by Meta, the bottom row for images created by Midjourney. P-values < 0.050 are marked in bold. Note: Reported p-values correspond to the analyses of covariance (ANCOVAs) controlling for the effects of the depicted disease, race, and age (not for analyses on sex differences in age).

Racial/Ethnical Differences in Age, Facial Expression, and Weight Across all 29 Diseases

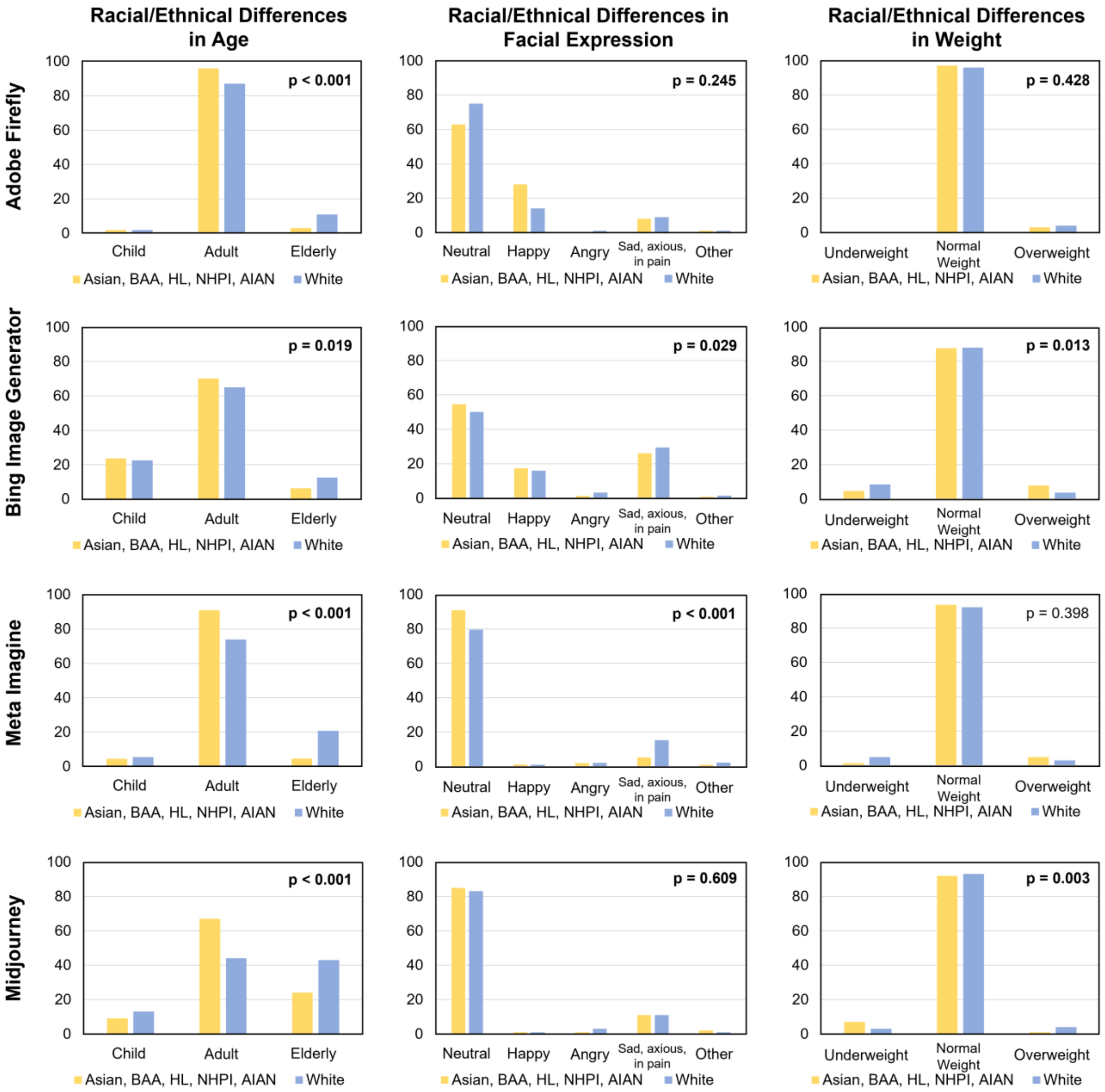

***Supplementary Figure 2*: Racial/ethnical differences in age (left), facial expression (middle), and weight (right) across all diseases**. The first row of graphs shows the racial/ethnical differences for images created by Adobe, the second row for images by Bing, the third row for images by Meta, the bottom row for images created by Midjourney. P-values < 0.050 are marked in bold. Note: Reported p-values correspond to the analyses of covariance (ANCOVAs) controlling for the effects of the depicted disease, sex, and age (not for analyses on sex differences in age). Abbreviations: BAA = Black or African American, HL = Hispanic or Latino, NHPI = Native Hawaiian or Other Pacific Islander, AIAN = American Indian or Alaska Native.

References

1. Garfield, K. & Sergent, S.R. Pyloric Stenosis. in *StatPearls* (StatPearls Publishing, Copyright © 2024, StatPearls Publishing LLC., Treasure Island (FL), 2024).
2. Mahapatra, S. & Amsbaugh, M.J. Medulloblastoma. in *StatPearls* (StatPearls Publishing, Copyright © 2024, StatPearls Publishing LLC., Treasure Island (FL), 2024).
3. Li, Z.Z.*, et al.* Global, regional, and national burden of gallbladder and biliary diseases from 1990 to 2019. *World J Gastrointest Surg* **15**, 2564-2578 (2023).
4. Banerjee, P., Jain, A., Kumar, U. & Senapati, S. Epidemiology and genetics of granulomatosis with polyangiitis. *Rheumatol Int* **41**, 2069-2089 (2021).
5. Mayeux, R. & Stern, Y. Epidemiology of Alzheimer disease. *Cold Spring Harb Perspect Med* **2**(2012).
6. Schmidt, R.*, et al.* [Sex differences in Alzheimer's disease]. *Neuropsychiatr* **22**, 1-15 (2008).
7. Li, X.*, et al.* Global, regional, and national burden of Alzheimer's disease and other dementias, 1990-2019. *Front Aging Neurosci* **14**, 937486 (2022).
8. Padala, S.A.*, et al.* Epidemiology, Staging, and Management of Multiple Myeloma. *Med Sci (Basel)* **9**(2021).
9. Rawla, P. Epidemiology of Prostate Cancer. *World J Oncol* **10**, 63-89 (2019).
10. Global, regional, and national incidence, prevalence, and years lived with disability for 354 diseases and injuries for 195 countries and territories, 1990-2017: a systematic analysis for the Global Burden of Disease Study 2017. *Lancet* **392**, 1789-1858 (2018).
11. McHugh, J.*, et al.* Prostate cancer risk in men of differing genetic ancestry and approaches to disease screening and management in these groups. *Br J Cancer* **126**, 1366-1373 (2022).
12. Alshaikhli, A., Killeen, R.B. & Rokkam, V.R. Hemophilia B. in *StatPearls* (StatPearls Publishing, Copyright © 2024, StatPearls Publishing LLC., Treasure Island (FL), 2024).
13. Hantsoo, L.*, et al.* Premenstrual symptoms across the lifespan in an international sample: data from a mobile application. *Arch Womens Ment Health* **25**, 903-910 (2022).
14. Zhu, L.*, et al.* Global burden and trends in female premenstrual syndrome study during 1990-2019. *Arch Womens Ment Health* (2024).
15. Abalos, E., Cuesta, C., Grosso, A.L., Chou, D. & Say, L. Global and regional estimates of preeclampsia and eclampsia: a systematic review. *Eur J Obstet Gynecol Reprod Biol* **170**, 1-7 (2013).
16. Saginala, K., Barsouk, A., Aluru, J.S., Rawla, P. & Barsouk, A. Epidemiology of Melanoma. *Med Sci (Basel)* **9**(2021).
17. Morgese, F.*, et al.* Gender Differences and Outcomes in Melanoma Patients. *Oncol Ther* **8**, 103-114 (2020).
18. Walton, C.*, et al.* Rising prevalence of multiple sclerosis worldwide: Insights from the Atlas of MS, third edition. *Mult Scler* **26**, 1816-1821 (2020).
19. Global, regional, and national burden of multiple sclerosis 1990-2016: a systematic analysis for the Global Burden of Disease Study 2016. *Lancet Neurol* **18**, 269-285 (2019).
20. Shi, D.*, et al.* Trends of the Global, Regional and National Incidence, Mortality, and Disability-Adjusted Life Years of Malaria, 1990-2019: An Analysis of the Global Burden of Disease Study 2019. *Risk Manag Healthc Policy* **16**, 1187-1201 (2023).
21. Global, regional, and national prevalence and mortality burden of sickle cell disease, 2000-2021: a systematic analysis from the Global Burden of Disease Study 2021. *Lancet Haematol* **10**, e585-e599 (2023).
22. Kato, G.J.*, et al.* Sickle cell disease. *Nat Rev Dis Primers* **4**, 18010 (2018).

23. Saeed, F.*, et al.* A Narrative Review of Stigma Related to Infectious Disease Outbreaks: What Can Be Learned in the Face of the Covid-19 Pandemic? *Front Psychiatry* **11**, 565919 (2020).
24. Mak, W.W.*, et al.* Comparative stigma of HIV/AIDS, SARS, and tuberculosis in Hong Kong. *Soc Sci Med* **63**, 1912-1922 (2006).
25. Mody, A.*, et al.* HIV epidemiology, prevention, treatment, and implementation strategies for public health. *Lancet* **403**, 471-492 (2024).
26. UNAIDS. Global HIV & AIDS statistics — Fact sheet. (2022).
27. Abdool Karim, S.S., Abdool Karim, Q., Gouws, E. & Baxter, C. Global Epidemiology of HIV-AIDS. *Infectious Disease Clinics of North America* **21**, 1-17 (2007).
28. Mahajan, A.P.*, et al.* Stigma in the HIV/AIDS epidemic: a review of the literature and recommendations for the way forward. *Aids* **22 Suppl 2**, S67-79 (2008).
29. Glaziou, P., Floyd, K. & Raviglione, M.C. Global Epidemiology of Tuberculosis. *Semin Respir Crit Care Med* **39**, 271-285 (2018).
30. WHO. Global Tuberculosis Report 2023. (2023).
31. Courtwright, A. & Turner, A.N. Tuberculosis and stigmatization: pathways and interventions. *Public Health Rep* **125 Suppl 4**, 34-42 (2010).
32. Global, regional, and national burden of hepatitis B, 1990-2019: a systematic analysis for the Global Burden of Disease Study 2019. *Lancet Gastroenterol Hepatol* **7**, 796-829 (2022).
33. Brown, R., Goulder, P. & Matthews, P.C. Sexual Dimorphism in Chronic Hepatitis B Virus (HBV) Infection: Evidence to Inform Elimination Efforts. *Wellcome Open Res* **7**, 32 (2022).
34. Cotler, S.J.*, et al.* Characterizing hepatitis B stigma in Chinese immigrants. *J Viral Hepat* **19**, 147-152 (2012).
35. Tao, Y.T.*, et al.* Global, regional, and national trends of syphilis from 1990 to 2019: the 2019 global burden of disease study. *BMC Public Health* **23**, 754 (2023).
36. Chen, T.*, et al.* Evaluating the global, regional, and national impact of syphilis: results from the global burden of disease study 2019. *Sci Rep* **13**, 11386 (2023).
37. Gilman, S.L. Disease and stigma. *The Lancet* **354**, SIV15 (1999).
38. WHO. COVID-19 epidemiological update – 16 February 2024. (2024).
39. CDC. COVID-19 Stats: COVID-19 Incidence,* by Age Group† — United States, March 1–November 14, 2020§. (2021).
40. WHO. WHO COVID-19 dashboard. (2024).
41. Kharroubi, S.A. & Diab-El-Harake, M. Sex-differences in COVID-19 diagnosis, risk factors and disease comorbidities: A large US-based cohort study. *Front Public Health* **10**, 1029190 (2022).
42. Bhanot, D., Singh, T., Verma, S.K. & Sharad, S. Stigma and Discrimination During COVID-19 Pandemic. *Front Public Health* **8**, 577018 (2020).
43. Committee on the Science of Changing Behavioral Health Social, N.*, et al.* in *Ending Discrimination Against People with Mental and Substance Use Disorders: The Evidence for Stigma Change* (National Academies Press (US), Copyright 2016 by the National Academy of Sciences. All rights reserved., Washington (DC), 2016).
44. Rössler, W. The stigma of mental disorders: A millennia-long history of social exclusion and prejudices. *EMBO Rep* **17**, 1250-1253 (2016).
45. Alonso, J.*, et al.* Association of perceived stigma and mood and anxiety disorders: results from the World Mental Health Surveys. *Acta Psychiatr Scand* **118**, 305-314 (2008).
46. Solmi, M.*, et al.* Age at onset of mental disorders worldwide: large-scale meta-analysis of 192 epidemiological studies. *Mol Psychiatry* **27**, 281-295 (2022).

47. Liu, Q., *et al.* Changes in the global burden of depression from 1990 to 2017: Findings from the Global Burden of Disease study. *Journal of Psychiatric Research* **126**, 134-140 (2020).
48. Labaka, A., Goñi-Balentziaga, O., Lebeña, A. & Pérez-Tejada, J. Biological Sex Differences in Depression: A Systematic Review. *Biol Res Nurs* **20**, 383-392 (2018).
49. McHugh, R.K., Votaw, V.R., Sugarman, D.E. & Greenfield, S.F. Sex and gender differences in substance use disorders. *Clin Psychol Rev* **66**, 12-23 (2018).
50. Degenhardt, L., Stockings, E., Patton, G., Hall, W.D. & Lynskey, M. The increasing global health priority of substance use in young people. *Lancet Psychiatry* **3**, 251-264 (2016).
51. Simha, A., *et al.* Effect of national cultural dimensions and consumption rates on stigma toward alcohol and substance use disorders. *Int J Soc Psychiatry* **68**, 1411-1417 (2022).
52. Javaid, S.F., *et al.* Epidemiology of anxiety disorders: global burden and sociodemographic associations. *Middle East Current Psychiatry* **30**, 44 (2023).
53. Solmi, M., *et al.* Incidence, prevalence, and global burden of schizophrenia - data, with critical appraisal, from the Global Burden of Disease (GBD) 2019. *Mol Psychiatry* (2023).
54. Cortese, S., *et al.* Incidence, prevalence, and global burden of ADHD from 1990 to 2019 across 204 countries: data, with critical re-analysis, from the Global Burden of Disease study. *Mol Psychiatry* **28**, 4823-4830 (2023).
55. Mueller, A.K., Fuermaier, A.B., Koerts, J. & Tucha, L. Stigma in attention deficit hyperactivity disorder. *Atten Defic Hyperact Disord* **4**, 101-114 (2012).
56. Puhl, R.M., Himmelstein, M.S. & Speight, J. Weight Stigma and Diabetes Stigma: Implications for Weight-Related Health Behaviors in Adults With Type 2 Diabetes. *Clin Diabetes* **40**, 51-61 (2022).
57. Maguire, R., *et al.* Lung cancer stigma: A concept with consequences for patients. *Cancer Rep (Hoboken)* **2**, e1201 (2019).
58. Vaughn-Sandler, V., Sherman, C., Aronsohn, A. & Volk, M.L. Consequences of perceived stigma among patients with cirrhosis. *Dig Dis Sci* **59**, 681-686 (2014).
59. Panza, G.A., *et al.* Links between discrimination and cardiovascular health among socially stigmatized groups: A systematic review. *PLoS One* **14**, e0217623 (2019).
60. Sørensen, T.I.A., Martinez, A.R. & Jørgensen, T.S.H. Epidemiology of Obesity. *Handb Exp Pharmacol* **274**, 3-27 (2022).
61. Haslam, D.W. & James, W.P.T. Obesity. *The Lancet* **366**, 1197-1209 (2005).
62. Blüher, M. Obesity: global epidemiology and pathogenesis. *Nat Rev Endocrinol* **15**, 288-298 (2019).
63. Puhl, R.M. & Heuer, C.A. The stigma of obesity: a review and update. *Obesity (Silver Spring)* **17**, 941-964 (2009).
64. Dai, H., *et al.* Global, regional, and national burden of ischaemic heart disease and its attributable risk factors, 1990-2017: results from the Global Burden of Disease Study 2017. *Eur Heart J Qual Care Clin Outcomes* **8**, 50-60 (2022).
65. Khan, M.A.B., *et al.* Epidemiology of Type 2 Diabetes - Global Burden of Disease and Forecasted Trends. *J Epidemiol Glob Health* **10**, 107-111 (2020).
66. Zhou, B., *et al.* Worldwide burden and epidemiological trends of tracheal, bronchus, and lung cancer: A population-based study. *EBioMedicine* **78**, 103951 (2022).
67. The global, regional, and national burden of cirrhosis by cause in 195 countries and territories, 1990-2017: a systematic analysis for the Global Burden of Disease Study 2017. *Lancet Gastroenterol Hepatol* **5**, 245-266 (2020).
